# Disparities in Early-Onset Colorectal Cancer

**DOI:** 10.3390/cells10051018

**Published:** 2021-04-26

**Authors:** Charles Muller, Ehizokha Ihionkhan, Elena M. Stoffel, Sonia S. Kupfer

**Affiliations:** 1 Section of Gastroenterology, Hepatology and Nutrition, University of Chicago, Chicago, IL 60637, USA; Charles.Muller@uchospitals.edu (C.M.); Ehizokha.Ihionkhan@uchospitals.edu (E.I.); 2 Division of Gastroenterology, University of Michigan, Ann Arbor, MI 48109, USA; estoffel@med.umich.edu

**Keywords:** disparities, early onset, colorectal cancer

## Abstract

The incidence and mortality of early-onset colorectal cancer (CRC) are increasing in the United States (US) and worldwide. In the US, there are notable disparities in early-onset CRC burden by race/ethnicity and geography. African Americans, Hispanic/Latinos, and populations residing in specific regions of the Southern U.S. are disproportionately affected with CRC diagnosed at younger ages, while less is known about disparities in other countries. Reasons for these disparities are likely multi-factorial and potentially implicate differences in health determinants including biology/genetics, diet/environment, individual health behaviors, and access to high-quality health services, as well as social and policy factors. This review summarizes current understanding of early-onset CRC disparities and identifies specific research areas that will inform evidence-based interventions at individual, practice, and policy levels to reduce the global burden of this disease.

## 1. Introduction

The incidence and mortality of colorectal cancer (CRC) have declined in the United States (US) since the late 1970s [1]. Much of this decrease is thought to be due to increased screening and improvements in treatment [2,3]. Yet while the overall burden of CRC has declined and survival gains have been made for older adults, there is a simultaneous and alarming increase in CRCs diagnosed in individuals younger than 50 years of age [4], which now account for roughly 10% of new CRC cases in the US [2].

The alarming rise in early-onset CRC is not limited to the US, as CRC diagnoses among individuals age <50 have increased in 19 out of 36 countries included in a global analysis [5]. Nine of these countries demonstrated increases in CRCs only among individuals younger than 50 years, while incidence was stable or declining in older populations [5]. Moreover, the timeframe for rising early-onset CRC is shared globally, with multiple countries experiencing rising incidence after 1990 [5]. Surveillance, Epidemiology and End Results program (SEER) demonstrated that the more recent rising incidence in the US was preceded by an initial decline early-onset CRC incidence, with incidence then increasing by 2% per year after 1990 [6]. This patterns suggests a birth cohort effect of shared risk factors for individuals born after 1960 [6].

Efforts to identify etiological genetic and environmental/behavioral risk factors, both of which contribute to an individual’s cancer risk, that could explain this striking trend in early-onset CRC incidence have been the subject of research and are a public health priority. Genetic risk factors contribute to the burden of early-onset CRC; for example, nearly one in five individuals diagnosed with CRC under age 50 was found to carry a pathogenic variant in a cancer-related gene [7,8]. Family CRC history is an established risk factor with an approximately 2-fold increased risk among first-degree relatives with recommendations to begin screening at age 40 [9]. However, one in four early-onset CRC patients who could have undergone earlier screening based on family history guidelines was not screened [10]. Despite these observations about genetic contributions to early-onset CRC, the fact that genetic risk factors do not change for a population over time suggests that the greater focus should be on generational differences in diet, lifestyle, or environmental risk factors that could contribute to early-onset CRC or their interplay with underlying genetic susceptibility.

In the US, it is notable that the burden of early-onset CRC falls disproportionately on minorities and individuals in specific geographic regions mirroring CRC disparities observed in older adults. Health disparities are defined as health outcomes that are greater or less between populations defined by age, race/ethnicity, sex, and geographic region among others. A number of health determinants are thought to contribute to health disparities and fall into five broad categories including biology/genetics, individual behavior, health services, social factors, and policies [11] (Table 1). The relationship between health determinants is complex, and addressing health disparities requires a multi-level approach. This article summarizes current understanding of disparities in early-onset CRC epidemiology, risk factors (environmental and genetic), tumor biology, and outcomes with emphasis on differences by age, race/ethnicity, and geography in the US and, when available, worldwide.

## 2. Early-Onset CRC Epidemiology and Disparities

Population-based studies have demonstrated a pattern of increasing incidence of CRC among individuals younger than 50 in the United States since 1975 [1,4,12], during which time CRC incidence increased 0.41% and 1.99% annually for individuals 20–34 and 35–49, respectively. Based on existing trends, incidence of colon and rectal cancer among individuals younger than 50 is expected to increase 90% and 124%, respectively by 2030 [4], at which point individuals younger than 50 will comprise 11% and 23% of colon and rectal cancer patients (compared to 4.8% and 9.5% in 2010). This trend seems to has accelerated since the 1990s, when age-adjusted CRC incidence rose from 7.9 per 100,000 to 11.2 per 100,000 among individuals aged 20-49 [13,14,15]. Furthermore, CRC incidence is rising fastest among younger individuals, with a 5.2% annual increase in incidence among ages 20–29, 3% for 30–39, and 1.5% for 40–49 [4,12,14].

**Figure 1:**
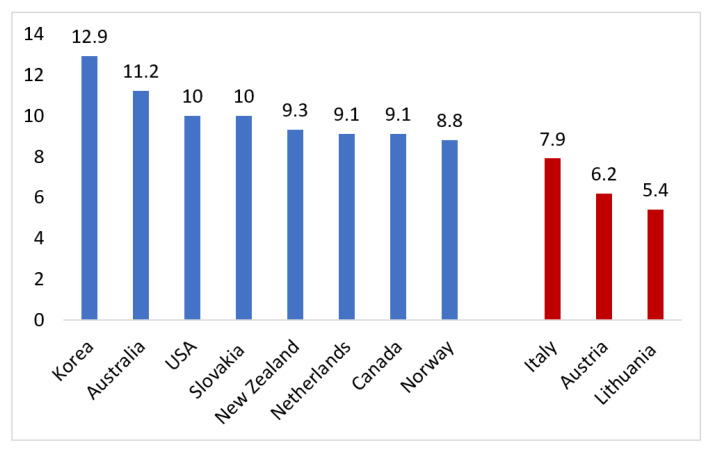
Age-standardized incidence rate during 2008–2021 for colorectal cancer among adults ages 20–49 years worldwide. Shading indicates trend in incidence rates based on 10-year average annual % change; blue, significant increase; red, significant decrease. The figure was recreated by using the incidence rates from Siegel et al. 2019 [5].

The rising incidence of early-onset CRC is not a phenomenon unique to the United States (Figure 1). Population-based studies have demonstrated rising incidence in many high-income nations with Western-influenced culture, [16] including Australasia [17,18,19], Western European nations, [20,21] Canada, [22,23], and South Korea [5]. A large study of global trends in CRC incidence in 36 countries found that 19 countries experienced increasing incidence of CRC among adults age 20–49 from 2008 to 2012 [5]. Nine of these countries, which were localized to North America, Western Europe, and Australasia, exhibited a pattern similar to the United States in which early-onset CRC rose amidst a backdrop of declining CRC incidence among adults older than 50 [5]. Korea, the country with the highest rise in early-onset CRC incidence over that span, also saw a sharp rise in CRC incidence for adults older than 50, which was possibly due to a more abrupt adoption of a Westernized diet over the past several decades [5]. Similar to Korea, other Asian and Middle Eastern countries have experienced a rise in CRC across all age groups, but with younger-age individuals constituting a larger and rising proportion of those at risk for CRC [24,25]. In East Asia, young-onset male rectal cancer in particular appears to be on the rise, as observed from 20 year data from Korea, Japan, Taiwan, and Hong Kong [26]. A similar trend has been observed in India, where the fastest growing CRC demographic is seen among men younger than 50 [27].

From 2008 to 2012, early-onset CRC incidence only saw a significant decrease in three countries (Italy, Austria, and Lithuania) during the study period. Interestingly, both Italy and Austria have initiated average-risk CRC screening for individuals at ages 40 and 44, respectively, for decades. In those countries, the declining early-onset CRC incidence was limited only to the 40–49 age groups, while incidence increased for those younger than 40 [5].

**Figure 2:**
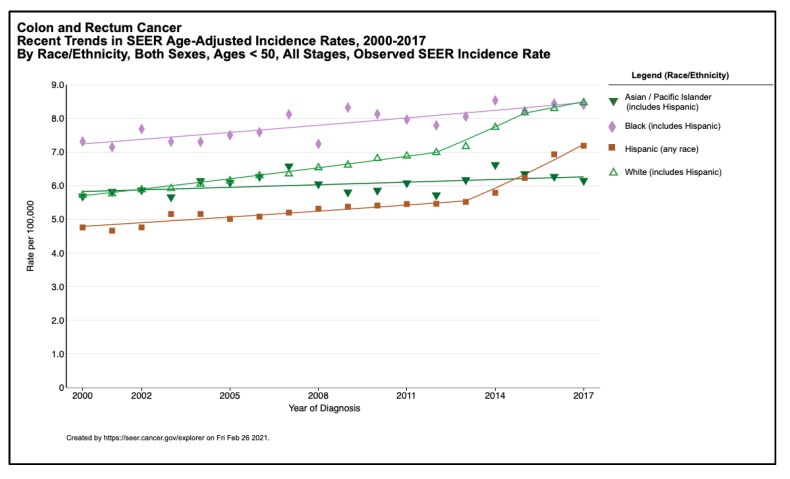
Observed incidence rates of colorectal cancer by race/ethnicity, both sexes, ages 20–49 years, United States, 2000–2017 from the SEER registry [39].

In the United States, African-Americans have long been known to have an increased burden of CRC compared to Whites, with higher incidence, worse outcomes, and earlier-onset of disease [1,28,29,30,31,32]. While for later-onset CRC, incidence overall is declining, the incidence for African-Americans, which was already higher than that of Whites, only decreased by 26% from 1975 to 2013, compared to 40% for Whites during the same period [33]. In addition to higher overall incidence of CRC, African-Americans have higher incidence of early-onset CRC compared to Whites (12.2 vs. 9.2 per 100,000) (Figure 2) [13,15,32]. Across all ages, African-Americans have a CRC incidence 1.28 times that of Whites [34]. However, by 2040, this ratio is expected to fall to 1.14 [34]. This expected reduction in the disparity of CRC incidence between Whites and African-Americans may be in part due to improved uptake of CRC screening among African-Americans older than 50 [35,36,37]. Despite the recommendation in the last decade to start screening African Americans at an earlier age than non-African Americans [9] and some preliminary evidence suggesting equivalent uptake among African Americans age 45–50 [38], there is not yet population-level data to suggest this recent recommendation is impacting early-onset CRC incidence among African Americans. The narrowed incidence gap more likely represents the increasing incidence of early-onset CRC among Whites, particularly for rectal cancer [13,34]. SEER data from 1992 to 2005 demonstrated that early-onset CRC incidence rose 2–2.2% annually among Whites, but it actually decreased 0.2–0.6% annually among African-Americans [14]. Similar small decreases in early-onset CRC incidence among African-Americans from 1990 to 2014 were demonstrated by California statewide data [37]. More recent nationwide data have shown early-onset CRC incidence rise from 7.5 to 11.0 per 100,000 in Whites compared to 11.7 to 12.7 per 100,000 for African-Americans by 2014 [13]. The increased pace of rising incidence of early-onset CRC among Whites is largely driven by rising rectal cancer incidence, which rose at triple the rate seen in African-Americans over the past two decades. In fact, Whites have now surpassed African-Americans in incidence of early-onset rectal cancer as of 2014, despite persistent disparities in CRC incidence overall [13].

Nationwide data has shown that similar to African-Americans, the proportion of Hispanic/Latinos diagnosed with CRC younger than age 50 is nearly double the rate seen in Whites (12% vs. 6.7%) (Figure 2) [32]. However, Hispanic/Latinos have lower overall CRC incidence than Whites across all age groups, including for individuals younger than 50 [40,41,42].

However, the incidence of early-onset CRC is rising at a faster rate among Hispanic/Latinos [32,37]. Siegel and colleagues demonstrated that Hispanic Latino men were among the fastest growing demographic of early-onset CRC, with an annual increase of 2.7% from 1992 to 2005 [14]. The incidence for Hispanic/Latina women, by contrast, only rose 1.2% annually during the same time [14]. Overall, incidence of early-onset CRC among Hispanic/Latinos has risen 2.35% annually, compared to 2.02% for Whites [32]. A similar trend was observed in analyses of statewide data from California and Texas [37,41].

**Figure 3:**
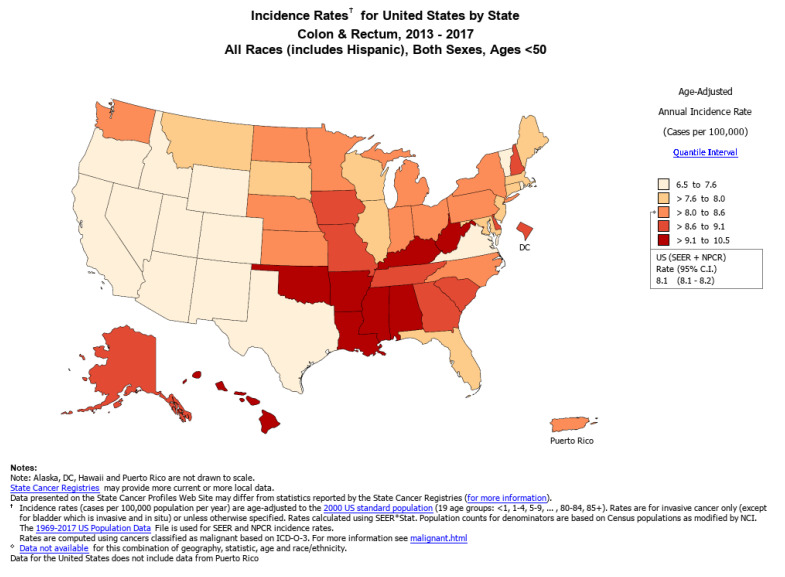
Incidence rates of early-onset CRC (Ages < 50) by US state, 2013–2017 from SEER registry [39].

Within the United States, early-onset CRC incidence is highest in southern states and Appalachia, [2] with Kentucky reporting the highest incidence of 14.3 per 100,000 individuals aged 20–49 between 1995 and 2015 (Figure 3) [43]. Another study of the National Cancer database from 1998 to 2007 showed that compared to CRC among adults older than 50, early-onset CRC was more prevalent in southern states than elsewhere in the United States [15]. Conversely, western states have demonstrated the lowest rates of early-onset CRC burden [2] but reported some of the highest relative increases in early-onset CRC incidence, which was primarily driven by increasing rectal cancer among Whites [43]. State-by-state early-onset CRC incidence has been stable overall among African-Americans and Hispanic/Latinos, while early-onset CRC incidence rose for Whites in 40 states from 1995 to 2015 [43].

## 3. Environmental/Lifestyle Risk Factors and Disparities

A number of environmental and lifestyle factors have been proposed as likely contributors to disparities in early-onset CRC (Table 2). Obesity, whether reported as body mass index (BMI), waist circumference, or visceral adiposity, is a well-established risk factor for CRC [44,45,46,47,48]. Therefore, rising incidence of obesity [49,50], particularly among young adults [51], has been suggested as an important potential mechanism for the rising incidence in early-onset CRC. In fact, recent work has demonstrated BMI to be a significant risk factor for early-onset CRC in a dose-dependent manner [52]. However, given mixed data on the effect of adulthood obesity on early-onset CRC [53,54,55], in addition to its shortened latency period, greater attention has been paid to childhood obesity, which has risen across all childhood age groups over the past three decades [56], as a potential mechanism for early-onset CRC [57]. For example, recent work has shown that obesity at age 8, but not at puberty, was associated with increased CRC risk [58]. BMI at age 18 and weight gain since age 18 have also both been demonstrated to be risk factors for early-onset CRC [52]. This association between childhood obesity and CRC appears to be more strongly linked to colon than rectal cancer [58,59,60]. The consistently higher prevalence of childhood obesity and extreme obesity among African-Americans and Hispanic/Latinos over the past several decades compared to Whites, in whom rising childhood obesity has been a more recent trend [56,61], could provide an explanation for existing disparities in early-onset CRC and the observed narrowing of incidence between African-Americans and Whites over time.

Type II diabetes, which has long been implicated as a risk factor for CRC [62,63,64] and has been on the rise among children and young adults [65], has been proposed as an additional risk factor for early-onset CRC. In fact, a Korean study found diabetes to be a risk factor for early-onset but not older-onset CRC [57]. Although young-onset type II diabetes is on the rise for all racial/ethnic groups, the consistently greater burden among African-Americans and the widening burden among Hispanic/Latinos compared to Whites over the past three decades could represent another potential risk factor for disparities in early-onset CRC [65]. Increased prevalence of the metabolic syndrome, which is highest among US Hispanic/Latinos and is characterized by insulin resistance and hyperinsulinemia [66], serves as a shared risk factor for multiple gastrointestinal cancers, and it could explain patterns of rising incidence of CRC and other gastrointestinal cancers as a whole among young Hispanic/Latinos [67].

Western-style diets, characterized by high consumption of red or processed meats and low fiber intake, have been implicated as a risk factor for older-onset CRC and could explain global patterns of differing CRC risk [68,69,70,71]. Effects of diet on metabolic factors such as insulin resistance or bile acid composition in addition to influence on chronic inflammation or gut dysbiosis have been postulated as mechanisms for the influence of diet on CRC pathogenesis [70,72,73,74]. For example, certain dietary components, such as red meat, iron, cholesterol, fat, and carbohydrates are considered pro-inflammatory, while others such as fiber, omega fatty acids, caffeine, tea, and several vitamins are have anti-inflammatory properties [75]. Therefore, diets with a greater balance of pro-inflammatory components compared to those with anti-inflammatory properties, such as Westernized diets, could contribute to CRC risk [75]. Given trends of worsening diet quality over time in the United States and among young individuals [76,77], dietary factors have been reasonably studied as a mechanism for rising early-onset CRC. In fact, recent work has demonstrated that the Western diet is a significant risk factor for young-onset advanced adenomas, particularly in the distal colon and rectum, the anatomic subsites, which are largely responsible for the rise in early-onset CRC [78]. Therefore, disparities of diet quality, which is influenced by both race and socioeconomic status [77], could contribute to observed trends in disparities for early-onset CRC. Although dietary factors do not explain long-standing higher incidence of early-onset proximal colon cancers among African Americans, trends of higher prevalence of poor quality diet, including more processed foods and lower intake of fiber, fruits, and vegetables, among African-Americans, along with the narrowing gap in diet quality with Whites [77], have mirrored the increased, but narrowing, burden of early-onset CRC among African-Americans. Furthermore, US Hispanic/Latinos have higher CRC incidence than Hispanic/Latinos in Latin American countries, suggesting that changes in CRC risk factors occurring after migration to the United States influence the rising incidence [67]. Assimilation to Westernized diet, with its resultant effects on obesity and development of metabolic syndrome [73], is a possible mechanism of environmental contribution to CRC risk upon migrating to the United States.

**Table 2:** Potential environmental risk factors for early-onset colorectal cancer and how these could contribute to disparities by race/ethnicity.

Factor	Potential Impact on Disparities	References
Obesity	Increased prevalence of childhood obesity and extreme obesity in AAs and Hispanics	[50,55]
Type 2 diabetes	Increased prevalence in AAs and HispanicsIncreased prevalence of metabolic syndrome in Hispanics	[59,60]
Western diet	Poorer quality diet in AAs	[70]
Sedentary lifestyle	Increased rates of television viewing and decreased physical activity among minority children	[55,74]

Increasingly sedentary behavior among adolescents and young adults has been proposed as another potential explanation for rising early-onset CRC [79,80]. Although public health campaigns have resulted in an increase in physical activity in the US in recent years, increased computer or desk-based work practices and passive behaviors such as television watching, have resulted in a rise of overall sedentary behaviors over the years [61,81]. Minority children are particularly vulnerable to this trend, exhibiting higher rates of television viewing and less physical activity than Whites [61]. An analysis of television-watching activity in the Nurses’ Health Study II demonstrated a dose–responsive risk for early-onset CRC with a relative risk of 1.12 and 1.69 for those watching 7–14 h and more than 14 h per week of television, respectively, even after controlling for obesity and physical activity [82]. This trend was more pronounced for rectal cancer [82].

Many of the previously mentioned lifestyle/environmental factors contribute both to racial and geographic disparities in early-onset CRC. Within the United States, early-onset CRC “hotspots” also have high rates of obesity, sedentary behavior, and underinsurance [83]. Although these hotspots also have higher minority populations, racial disparities in incidence and outcome of early-onset CRC persist within hotspots, suggesting an interplay between race and lifestyle factors [83]. Further studies examining the impact of well-described lifestyle risk factors for early-onset CRC by race/ethnicity are needed.

Additional social and environmental factors and their interactions contributing to disparities in early-onset CRC are less well-understood. For example, a lack of insurance or public insurance have been found to be associated with a four-fold risk of early-onset CRC [15], while minority early-onset CRC patients are significantly more likely than their White counterparts to be underinsured [84]. The influence of insurance status on CRC is significantly influenced by income and socioeconomic status and confounded by many of the aforementioned lifestyle and environmental factors associated with early-onset CRC. Additionally, the influence of emerging evidence for antibiotic exposure [85,86,87], oral hygiene [88], psychological stress [89], and immune response/inflammation [90], all of which interplay with gut microbiota, in addition to traditional CRC risk factors such as alcohol [91], tobacco [92], and NSAIDs [93] on disparities in early-onset CRC has not yet been established.

## 4. Genetic Risk Factors and Disparities

Recent studies have estimated that 16–20% of individuals diagnosed with CRC age <50 harbor pathogenic germline variants in cancer susceptibility genes [7,8] compared to 10% of unselected CRC patients overall [94]. The prevalence of hereditary cancer syndromes may be even higher among younger individuals within that cohort [95]. Approximately half of the pathogenic germline variants identified in early-onset CRC occur in the mismatch repair (MMR) genes associated with Lynch syndrome [7,8]. Of note, at least one-third of early-onset CRC patients harboring pathogenic germline variants in cancer predisposition genes fail to meet classic genetic testing criteria for the identified gene [7,95], which led to the recommendation to perform germline multigene panel testing for all individuals diagnosed with CRC younger than age 50 [96]. It is worth noting that most studies reporting germline variants in the early-onset CRC population have been performed in primarily White populations, limiting our understanding of the mutation spectrum in minority populations [7,8], in whom hereditary cancer syndromes such as Lynch syndrome remain underdiagnosed [97].

Disparities in the referral and uptake of genetic testing in CRC patients of all ages pose an additional barrier to understanding the genetic contribution to early-onset CRC [98]. Indeed, recent work has demonstrated biased referral patterns of African-American and Hispanic/Latino early-onset CRC patients, who were significantly less likely to be referred for genetic evaluation compared to White patients [99]. Given that universal tumor testing for DNA mismatch repair deficiency (MMRd) to screen for Lynch Syndrome is performed at similar rates among minority populations and Whites, with similar rates of MMRd and/or germline mutation identification when downstream testing is actually performed [98,99], there is a clear need to implement systems that will facilitate universal multigene panel testing for all early-onset CRC patients, which may help close the gap in understanding the role of genetic susceptibility in early-onset CRC across different racial and ethnic groups.

Family history of CRC, which is reported by in 20–30% of CRC patients [100], is one of the most important risk factors for CRC and serves as the principal means by which individuals are stratified to undergo early screening in current practice [9]. A case-control study of risk factors for early-onset CRC found having a first degree relative (FDR) or a sibling with CRC were stronger predictors of CRC than any environmental or lifestyle factor, with odds ratios of 4 and 11 for FDR and siblings, respectively [54]. Previous studies have shown that both African-Americans [101] and Hispanic/Latinos [102] are less likely than Whites to have knowledge of their family history. A survey-based study out of California found that African-Americans were less likely than Whites to report a family history of CRC, which is at odds with observed increased incidence of CRC among African-Americans [103]. Therefore, lack of family history knowledge could lead to reduced uptake of otherwise indicated high-risk screening [102,104] and contribute to disparities in early-onset CRC incidence, particularly among those aged 40–49, which constitutes the largest proportion of early-onset CRC patients [32]. Although previous estimates have shown that only approximately 25–30% of early-onset CRC patients have a family history of CRC, which is similar to the rate seen in older-onset CRC [8,105], a recent large study found that early-onset CRC patients were nearly 3 times as likely to have a family history of CRC [106], highlighting that further work is needed to understand the influence of family history on early-onset CRC to allow for risk stratification and earlier identification of those at risk for early-onset CRC.

## 5. Tumor Characteristics and Disparities

Early-onset CRC tumors exhibit characteristics that differentiate them from sporadic older-onset CRCs, including predominance of distal tumors, poor differentiation, signet ring cells, lymphovascular, and perineural invasion [107,108]. Early-onset CRCs are less likely than older-onset tumors to have *BRAF* mutations [109,110] and have higher rates of *CTNNB1* mutations in very young patients [109]. Although previous studies have suggested lower rates of *APC* mutations among young-onset CRC tumors [109,110], a recent study using novel sequencing techniques to target a larger spectrum of known *APC* mutations [111] has suggested that *APC* mutations may be more common in early-onset CRC than previously thought. Molecular sub-typing of early-onset CRC tumors shows higher prevalence of CMS1 tumors in individuals less than age 40 associated with microsatellite instability, CIMP, hypermethylation, and immune infiltration [109]. Hypomethylation of LINE-1 elements also appears to be more frequent in tumors from patients under age 50 [112], suggesting unique methylation patterns in early-onset CRC. A multi-omics study demonstrated a unique signature in oxidative stress response in early-onset CRC [113].

Studies have evaluated tumor phenotype differences between African-Americans and Whites (reviewed in Carethers [114]); however, few of these have also considered molecular differences stratified by patient age (Table 3). Overall, African-Americans are more likely than Whites to have tumors in the proximal colon (although younger African-Americans patients were more likely than older individuals to have distal tumors [115], which is similar to trends noted in the overall population). Whole-genome somatic sequencing studies of CRC from African-American patients in Cleveland and Chicago found unique potential driver mutations in *EPHA6* [116], *FLCN* [116], and *CDH5* [110], although findings have not been replicated across studies. Notably, the study from the Chicago Colorectal Cancer Consortium [110] found that *APC*-negative tumors from African-Americans occurred at significantly younger ages (51.5 vs. 63 years), had lower overall mutation burden, fewer copy number variants, and distinct DNA methylation patterns including the hypermethylation of genes that regulate WNT signaling. Overall, early-onset CRCs have lower frequency of *APC* mutations [109], but whether additional findings from this study are unique to tumors from African-Americans remains to be determined.

Tumors from African-Americans are reported to have 20% lower odds of microsatellite instability [117] as well as impaired immune surveillance [118], which could contribute to worse prognosis. Rectal tumors from African-Americans have also been reported to have higher frequencies of an aggressive tumor-associated biomarker called elevated microsatellite alterations at selected tetranucleotide repeats or EMAST, which could also contribute to worse clinical outcomes [119]. Epigenetic age acceleration has been reported to differ by anatomic location between African-Americans and Whites with greater acceleration and unique methylation patterns in the right colon among African-Americans, which could contribute to differential cancer risks [120]. Whether these reported population differences in microsatellite instability, immune surveillance, EMAST, and methylation also extend to early-onset CRC remains to be studied.

Tumor phenotyping studies are largely lacking for other populations in the United States as well as for minority populations worldwide especially for early-onset CRC. One study from Puerto Rico reported overall lower rates of microsatellite instability, CIMP-high as well as *KRAS* and *BRAF* mutations compared to Whites from TCGA; however, it remains to be determined if these or other unique tumor phenotypes are found in other US Hispanic/Latino patients. Little is known about tumor phenotypes among Alaska Natives, who have the highest incidence of CRC in the United States, though it appears that rates of microsatellite instability are similar to other populations [121].

Worldwide, there are few molecular studies in minority populations with very few focused on early-onset CRC. One study from Brazil found overall similar rates of common somatic CRC mutations with a few notable differences based on African and Native American ancestry [122]. A study from South Africa noted younger age of diagnosis and higher rates of mucinous tumors in African patients compared to Indian, White, and mixed race patients [123]. A study of Maori, Polynesian, and White New Zealander CRC patients found differences in anatomic location as well as stage of presentation, although rates of CRC were lower among non-White individuals overall [124]. A study comparing characteristics of early-onset CRC in African-American and Nigerian patients noted an eight-fold increased rate of rectal tumors and earlier age of diagnosis among Nigerian patients, although no differences were noted by sex or histologic subtypes [125]. Worldwide, lack of data on ancestry and ethnicity significantly limits studies of differences in tumor characteristics and outcomes among minority populations. The unique features of CRC tumors among individuals with differing age, race, and ethnicity may reflect differences in the molecular drivers of carcinogenesis, which may have implications for treatment and preventions.

## 6. CRC Outcomes and Disparities

Studies of disparities in CRC survival consistently demonstrate lower survival among African-Americans, compared to Whites, which is a trend that appears to carry over to young CRC patients. Using SEER data from 2000 to 2009, Holowatyj and colleagues noted significantly lower 5-year survival for African-American/Black compared to White and Hispanic/Latino early-onset CRC patients (54.9% vs. 68.1% and 62.9%, respectively, *p* < 0.001) with a hazard ratio for cancer-specific death for African-Americans compared to Whites of 1.35 [126]. Using the National Cancer Database (NCBD), Sineshaw and colleagues studied CRC patients aged 18–64 from 2004 to 2012 and noted that insurance status and tumor characteristics accounted for 54% and 27% of excess deaths among African-American patients, suggesting that differences in oncologic care may contribute to differences in survival among young CRC patients [127]. Highlighting the role of geographic hot-spots for early-onset CRC (52% of which are in the south), Holowatyj and colleagues noted that individual and community-level factors accounted for approximately 1/3 of the variation early-onset CRC survival among women [128].

Studies have also noted disparities in early-onset CRC survival between proximal colonic and rectal tumors. Using SEER data from 1992 to 1996 and 2010 to 2014, Murphy and colleagues found that African-American early-onset CRC patients had smaller increases in survival for proximal tumors but increased survival for rectal tumors [13]. In 2010–2014, compared to Whites, survival disparities for African-Americans with proximal tumors had increased but had disappeared for rectal tumors during this same time period, suggesting that treatment and therapeutic responses play an important role in outcome disparities. Using data from NCBD, Alese and colleagues [129] found that despite comparable standard of care treatment, African-American early-onset CRC patients had lower 5-year survival compared to Whites and Hispanic/Latinos. The authors hypothesized that this survival disparity could have been due to more proximal cancers among African-Americans in this cohort and possible differences in tumor biology, which might impact risk of tumor recurrence and/or response to conventional oncologic treatments.

## 7. Conclusions

In 1966, Martin Luther King declared, “Of all the forms of inequality, injustice in health care is the most shocking and inhumane.” [130]. Unfortunately, health disparities, including in the diagnosis and treatment of early-onset CRC, persist and, in some cases, are widening. To date, disparities by race/ethnicity and, to a lesser extent, geographic location in outcomes of early-onset CRC suggest that biology/genetics, individual health behaviors, and access to and utilization of health services likely all have a role. Other social factors such as systemic racism, chronic stress, and neighborhood deprivation also deserve more rigorous investigation. Ultimately, this research will help inform evidence-based interventions in areas of biology and healthcare policy and practice to reduce the global burden of early-onset CRC.

## Figures and Tables

**Table 1:** Domains of determinants of health with examples. Adapted from Healthy People 2020 [11].

Domain	Examples
Biology/Genetics	Age; sex; inherited condition; family history
Individual behavior	Diet; physical activity; habits (e.g., tobacco, alcohol)
Health services	Lack of or limited access to healthcare; lack of insurance
Social factors	Job; discrimination; exposure to crime; social support; socioeconomic status; neighborhood deprivation; quality schools; transportation; public safety; residential segregation; exposure to environmental toxins
Policies	Local, state, and federal policies affecting health

**Table 3:** CRC tumor characteristics in African-American vs. White patients. In most cases, these characteristics have not been studied specifically in the context of early-onset colorectal cancer unless otherwise noted.

Characteristic	Details for African-American patients	References
Anatomic location	Overall more proximal vs. distal tumorsYounger AAs have higher prevalence of distal tumors vs. older AAs	[107]
Somatic mutations	Unique mutations in *EPHA6, FLCN* and *CDH5APC*-negative tumors more frequently in younger AA	[102,103,108]
Microsatellite instability	20% lower rate of microsatellite instabilityHigher rate of EMAST in rectal tumors	[109,110,111]
Epigenetics	Unique pattern of epigenetic signature in proximal colon	[112]

AA, African American; EMAST, elevated microsatellite alterations at selected tetranucleotide repeats.

## Data Availability

Not applicable.
